# Applications of Metformin in Dentistry—A review

**DOI:** 10.1016/j.jtumed.2023.03.014

**Published:** 2023-04-11

**Authors:** Muhammad Khawaja Hammad Uddin, Muhammad Shahrukh Khan Sadiq, Ashfaq Ahmed, Mariam Khan, Tooba Maniar, Syeda Mamoona Mateen, Bilquees Saba, Syed Muhammad Kashif, Shumaila Usman, Shariq Najeeb, Zohaib Khurshid, Muhammad Sohail Zafar

**Affiliations:** a Department of Science of Dental Materials, Dr. Ishrat-ul-Ebad Khan Institute of Oral Health Sciences, Dow University of Health Sciences, Karachi, Sindh, Pakistan; b Department of Oral Pathology, Bahria University Dental College, Bahria University Health Sciences Campus (Karachi) Karachi, Sindh, Pakistan; c Dr Panjwani Center for Molecular Medicine and Drug Research, International Center for Chemical and Biological Sciences, University of Karachi, Sindh, Pakistan; d Evidentia Dental Outcomes Research, Calgary, Alberta, Canada; e Schulich Dentistry, Schulich School of Medicine and Dentistry, Western University, London, ON N6A 5C, Canada; f Department of Medicine, Ziauddin Medical College, Ziauddin University, Karachi, Sindh, Pakistan; g Department of General Medicine, Civil Hospital, Dow University of Health Sciences, Karachi, Sindh, Pakistan; h Department of Molecular Medicine, Ziauddin Medical College, Ziauddin University, Karachi, Sindh, Pakistan; i Department of Prosthodontics and Dental Implantology, King Faisal University, Hofuf, Al-Ahsa, Saudi Arabia; j Center of Excellence for Regenerative Dentistry, Department of Anatomy, Faculty of Dentistry, Chulalongkorn University, Bangkok 10330, Thailand; k Department of Restorative Dentistry, College of Dentistry, Taibah University, Al Madina Al Munawara, 41311, Saudi Arabia; l Department of Dental Materials, Islamic International Dental College, Riphah International University, Islamabad 44000, Pakistan; m School of Dental Care Professionals (SDCP), Dow University of Health Sciences Karachi, Sindh, Pakistan

**Keywords:** ميتفورمين, التهاب اللثة, الخلايا الجذعية, سرطان الخلايا الحرشفية, اندماج عظمي, تقويم الأسنان, أنظمة توصيل الأدوية, Metformin, Periodontitis, Carcinoma, Squamous cell, Stem cells, Osseointegration, Orthodontics, Photochemotherapy

## Abstract

Metformin is a versatile drug with numerous medical uses. It is known primarily as an anti-hyperglycemic drug that has become the main oral blood-glucose-lowering medication for managing type 2 diabetes mellitus globally. Its use has been reported in a variety of oral conditions and dentistry in general. Recent clinical trials have indicated the effectiveness of adjunct topical application of metformin in improving the periodontal parameters of patients with diabetes and periodontitis. Additionally, studies have suggested that metformin stimulates odontogenic differentiation and mineral synthesis of stem cells in the tooth pulp. Metformin also stimulates osteoblast proliferation, decreases osteoclast activity and exerts regenerative effects on periodontal bone, thus making it a viable candidate for periodontal regeneration. Metformin monotherapy significantly enhances osseointegration of endosseous implants and has been reported to have anti-cancer effects on oral squamous cell carcinoma by impeding tumor progression. Animal studies have indicated that metformin improves orthodontic tooth movement and resists orthodontic appliance corrosion. This narrative review aims to provide a current summary of research highlighting the prospective uses of metformin in dentistry.

## الملخص

الميتفورمين دواء متعدد الاستخدامات وله استخدامات عديدة في مجال الطب. في المقام الأول، يُعرف بأنه عقار مضاد لارتفاع سكر الدم، وقد أصبح الدواء الأول عن طريق الفم لخفض نسبة الجلوكوز في الدم لعلاج داء السكري من النوع الثاني على مستوى العالم. توضح الأدبيات الحالية أيضا استخدام الميتفورمين في مجموعة متنوعة من أمراض الفم وطب الأسنان بشكل عام. أفادت التجارب السريرية الحديثة عن فعالية التطبيق الموضعي المساعد للميتفورمين في تحسين معايير اللثة لمرضى السكري المصابين بالتهاب دواعم السن. بالإضافة إلى ذلك، أشارت الدراسات إلى أنه يضفي تمايزا سنيا وكذلك تخليقا معدنيا للخلايا الجذعية في لب الأسنان. كما أنه يحفز تكاثر بانيات العظم ويقلل من نشاط ناقضات العظم مع الميل إلى ممارسة تأثير تجديدي على عظم دواعم السن مما يجعله مرشحا حيويا لتجديد اللثة. كما يعزز العلاج الأحادي بالميتفورمين الاندماج العظمي للغرسات الداخلية بشكل كبير. وذكرت بعض الدراسات أيضا أن له آثارا مضادة للسرطان على سرطان الخلايا الحرشفية عن طريق إعاقة تطور الورم. كما توض1ح بعض الأدبيات أيضا تأثيره الإيجابي في تحسين حركة الأسنان التقويمية ومقاومة تآكل أجهزة تقويم الأسنان في النماذج الحيوانية. تهدف هذه المراجعة السردية الحالية إلى تقديم ملخص حديث للبحوث العلمية حول الاستخدامات المحتملة للميتفورمين فيما يتعلق بمجال طب الأسنان.

## Introduction

### Background

**Figure 1:**
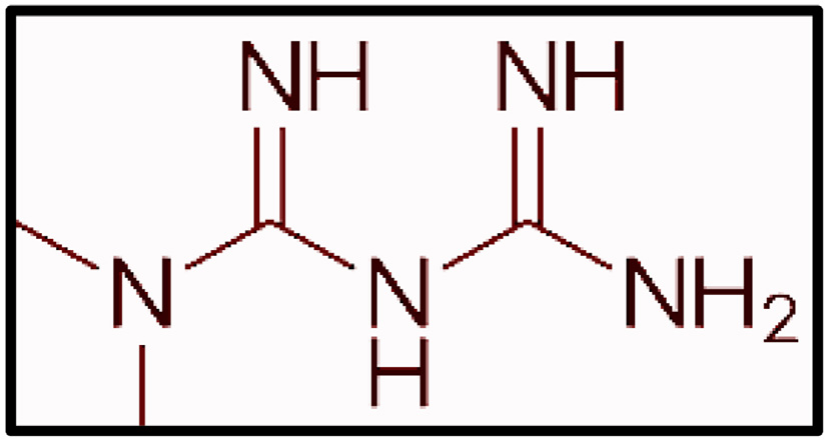
Molecular structure of metformin.

Metformin (1, 1-dimethyl biguanide; MF) is a renowned drug with versatile utility. MF is an orally administered anti-hyperglycemic drug and is the preferred first-line drug for lowering blood glucose levels in patients with type 2 diabetes mellitus (T2DM).^1^ MF, a second-generation biguanide, is extracted from the French lilac (*Galega officinalis*). In the Middle Ages, this plant extract was used to decrease blood sugar, relieve intense urination and alleviate symptoms of T2DM.^2^ In 1922, guanidine was discovered as an active ingredient in *G. officinalis* that lowers blood glucose levels.^3^ MF was overshadowed by phenformin and buformin—other guanide-derived drugs that were initially deemed more effective in treating T2DM. However, because of the high toxicity of the other medications, MF became the gold standard drug for managing high glucose levels in patients with diabetes, owing to its benign, nontoxic nature.^4^ The drug was approved for treating hyperglycemia in 1958 in the United Kingdom, in 1972 in Canada and in 1995 in the United States.^5^ MF is the most prescribed drug for T2DM management worldwide and is used by more than 120 million people.^6^ The molecular structure of MF is illustrated in Figure 1.

### Routes of administration

As an orally administered drug, MF decreases glucose levels in the blood by inhibiting gluconeogenesis in the liver.^7^ This decrease is achieved by inhibition of intracellular binding of calcium in hepatocyte mitochondria, thus decreasing gluconeogenesis.^8^ Moreover, MF is the principal therapeutic drug for the treatment T2DM in obese patients.^2^ Beyond its antidiabetic effects, MF has antilipidemic, hepatoprotective, anti-neoplastic, cardioprotective and anti-obesity effects.^9^ It has various applications including the management of acne, hirsutism and polycystic ovarian syndrome, and as a chemo-preventive agent for neoplastic conditions.^10^

### Pharmacological properties

After oral administration, MF is gradually absorbed from the small intestine. The bioavailability of the drug has found to be approximately 50–60%, and the plasma half-life has been calculated to be 1.5–4 h.^11^ Approximately 30–50% of an orally consumed dose of MF is excreted in the urine within the first 24 h, whereas 30% is unchanged and excreted via the feces.^12^ Importantly, MF can pass through the placenta, and fetal concentrations of MF are often lower than maternal concentrations of MF. The pharmacokinetics of MF in pregnant women is influenced by the high glomerular filtration rate. The plasma concentrations of MF during pregnancy are therefore lower than those in non-pregnant women.^13^

### Mode of action and subsequent effects

**Figure 2:**
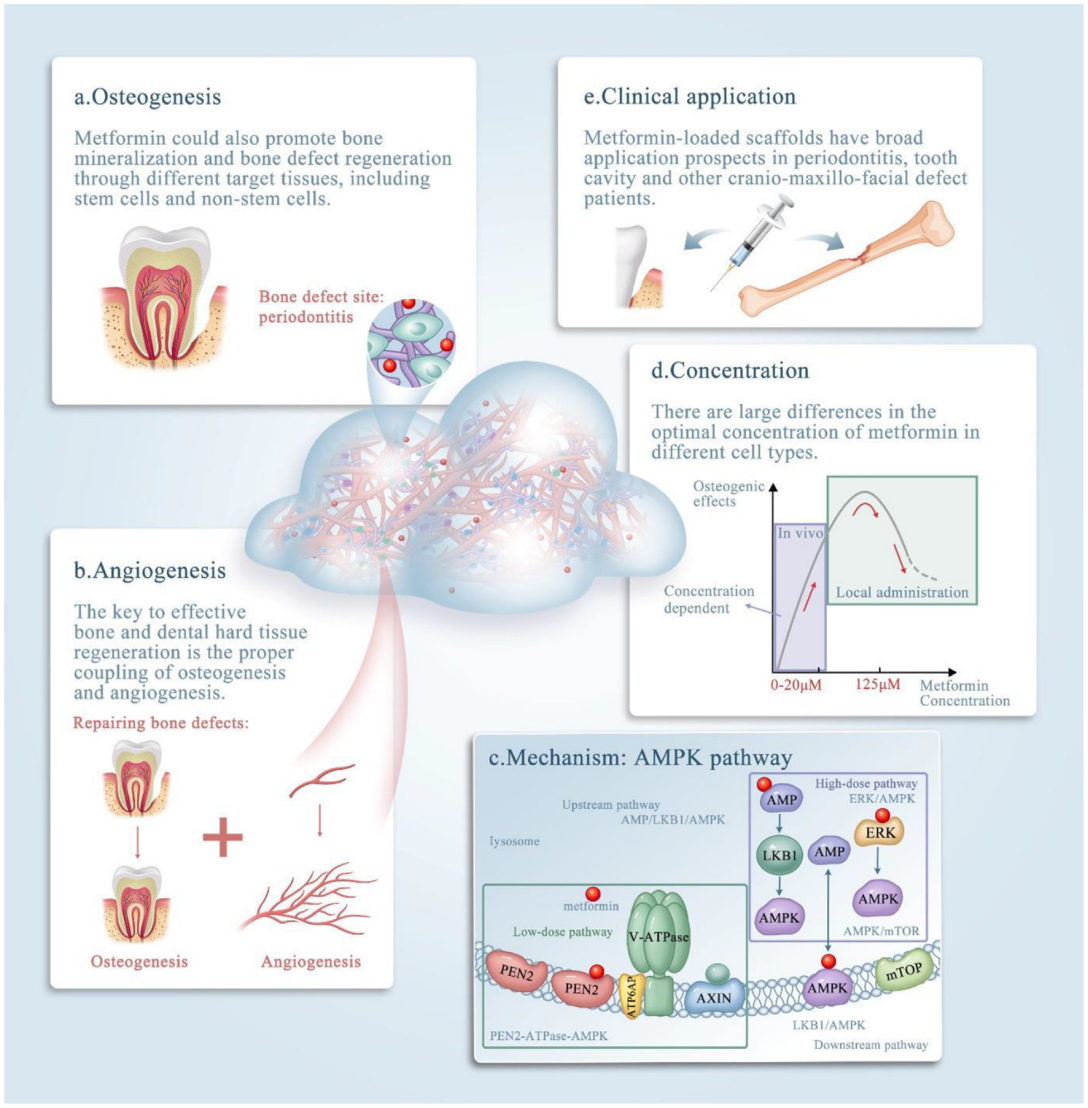
Effects of metformin in bone tissue engineering and clinical applications may contribute to the diagnosis and treatment of bone defects. (**a**–**e**) Schematic illustrating metformin delivery via biomaterials, mainly via scaffolds, and its effects on bone and dental tissue engineering. Note that metformin exerts its effects by (**a**) enhancing osteogenesis, (**b**) enhancing angiogenesis, (**c**) affecting the AMPK pathway and (**d**) acting in a concentration-dependent manner. (**e**) The clinical applications of metformin.^88^

MF exerts anti-obesity effects by decreasing appetite and increasing secretion of glucagon-like peptide-1 (GLP-1). The anti-hyperglycemic effect of MF decreases intestinal carbohydrate absorption (decreased postprandial hyperglycemia). The inhibition of hepatic gluconeogenesis occurs by halting of the Krebs cycle and oxidative phosphorylation after activation of AMP-activated protein kinase (AMPK).^14^ The promotion of glucose transport in skeletal muscle stimulated by insulin enhances the function of glucose transporter type 4 (GLUT-4) and increases the non-oxidative glucose disposal in skeletal muscle.^15^ Additionally, MF exerts anti-lipidemic effects through increasing free fatty acid esterification and inhibiting lipolysis in adipose tissue. Furthermore, the action of MF protects β-cells against toxicity and lipotoxicity of glucose and preserves β-cell secretory capacity, thereby slowing the progression to severe diabetes.^16^ During long term use of MF, the decrease in hepatic insulin resistance and beneficial effects on lipid levels have hepatoprotective effects.^17^ MF also has indirect and direct anti-neoplastic effects. It exerts an indirect effect via decreasing insulin resistance and insulin-like growth factor 1 (IGF-1) levels. In contrast, MF exerts direct anti-neoplastic effects through AMPK-associated and AMPK-independent cellular pathways.^18^ The beneficial cardioprotective effects arise from the combined effects of a decrease in weight and an ameliorated lipid profile after long-term use of MF.^19^ Recent research has revealed that this drug also affects bone metabolism, influencing osteoblast and osteoclast differentiation via stimulating osteoprotegerin (OPG), and decreasing receptor activator of nuclear factor κB ligand (RANKL) expression.^20^ The mode of action of MF relevant to dentistry is shown in Figure 2.

## Applications of metformin in dentistry

**Figure 3:**
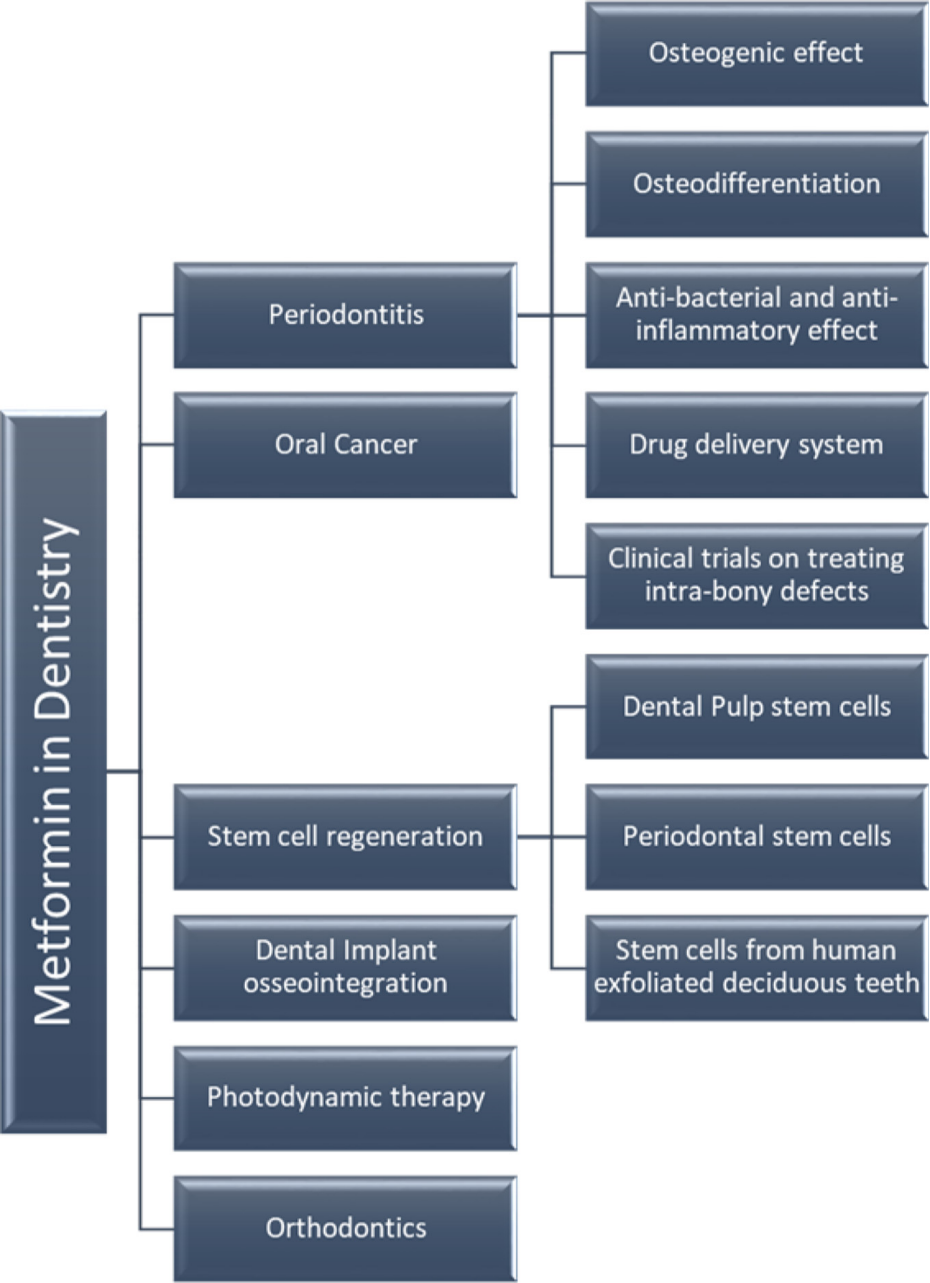
Applications of Metformin in Dentistry.

Numerous studies have demonstrated that MF can be used in various applications in dentistry (Figure 3), including in periodontitis, oral cancer, dental implants, dental stem cells, photodynamic therapy and orthodontics. This narrative review aims to provide a current summary of research highlighting the potential uses of MF in dentistry.

## Role of metformin in periodontitis

According to the European Federation of Periodontology, the term periodontitis refers to “a chronic inflammatory disease that is triggered by bacterial microorganisms and involves a severe chronic inflammation that causes the destruction of the tooth-supporting apparatus and can lead to tooth loss.“ 2^21^ It is characterized by a severe infectious-inflammatory response to poor oral hygiene.^22^ Despite the availability of numerous therapeutic and non-therapeutic treatment modalities, the prevalence of periodontitis is considerably high and affects most of the global population.^23^

### Osteogenic effects in periodontitis

At the *in vivo* and *in vitro* levels, MF has shown favorable effects in attenuating the destructive outcomes of diabetic periodontitis.^24^ The beneficial outcomes of using MF in periodontal conditions arise from its role in bone formation and remodeling. The mechanism of action underlying MF's osteogenic effect is binary, comprising an increase in osteoblast proliferation and a subsequent decrease in osteoclast activity.^25^^,^^26^ RANKL, receptor activator of nuclear factor κ B (RANK) and osteoprotegerin (OPG) form a system with a key role in the molecular regulation of bone remodeling.^27^ MF downregulates RANKL production while positively regulating the production of OPG from osteoblasts. Induction of bone formation and inhibition of resorption are caused by the negative regulation of osteoclast activity through a decreased RANKL/OPG ratio.^28^

### Osteogenic differentiation in periodontitis

In addition to its positive osteogenic effect, MF activates the osteogenic differentiation of a variety of cells via AMPK and mitogen-activated protein kinase (MAPK) signaling pathways.^25^^,^^29^^,^^30^ The outcome of AMPK signaling in promoting nodule formation and bone mass via osteoblastic differentiation has a crucial role in maintaining normal bone physiology. Cortizo et al. have revealed that MF significantly promotes mineralization in osteoblasts by activating extracellular signal regulated kinase (ERK) and the induction of endothelial and inducible nitric oxide synthases (e/iNOS). MF treatment of osteoblast mimicking cells (UMR106 and MC3T3E1) has been found to dose-dependently increase proliferation, osteoblastic differentiation and type-I collagen production in both cell lines, and to promote alkaline phosphatase (ALP) activity in MC3T3E1 osteoblastic cells.^25^ Along with AMPK activation, MF increases osteogenic differentiation of bone marrow progenitor cells via Runt-related transcription factor 2/core-binding factor α1 (Runx2/Cbfa 1). MF significantly decreases intracellular reactive oxygen species and apoptosis, and subsequently exerts a direct osteogenic effect on osteoblasts, which is mediated partially via promotion of Runx2 and insulin-like growth factor-1 expression (IGF-1).^29^

### Anti-bacterial and anti-inflammatory effects in periodontitis

An *in vitro* study has indicated that NLR family pyrin domain containing 3 (NLRP3) mediated pyroptosis plays a major part in diabetes-associated periodontitis. Cell death by pyroptosis is a major cause of severe inflammation in periodontitis due to T2DM. MF treatment has been found to attenuate pyroptotic death by inhibiting the NIMA-associated kinase 7/NLR family pyrin domain containing 3 (NEK7/NLRP3) pathway.^31^ In another recent study in human periodontal ligament cells stimulated with periopathogenic bacteria, a key inflammatory mediator, *Porphyromonas gingivalis* lipopolysaccharide (LPS), has been found to elicit the conditions existing in periodontitis.^32^ MF treatment significantly suppresses the inflammatory responses induced by *P. gingivalis* LPS in periodontal ligament cells, as characterized by diminished production and secretion of interleukin-1 beta (IL-1β) and interleukin-18 (IL-18).^32^ MF treatment also significantly decreases nucleotide-binding domain, leucine-rich-containing family, pyrin domain-containing-3 (NLRP3) and caspase-1 in human periodontal ligament cells.^32^

Moreover, MF has been reported to decrease bone loss in periodontal diseases. MF regulates nuclear factor kappa-light-chain-enhancer of activated B cells p65 (NF-κB p65) and high mobility group box 1 (Hmgb1) gene expression, thereby decreasing malondialdehyde, tumor necrosis factor-alpha (TNFα) and interleukin-1 beta (IL-1 β) levels, and subsequently decreasing bone loss in a rat model of periodontitis.^27^ In addition to countering bone loss, MF has positive effects in the healing of gingival wounds. In this regard, a recent study by Kaminato and colleagues has demonstrated that MF accelerates wound healing via Akt phosphorylation of fibroblasts in gingiva of insulin-resistant prediabetes animal models. Moreover, MF accelerates gingival wound healing in prediabetic mice by promoting the proliferation and migration of cultured human gingival fibroblasts *in vitro* via Akt phosphorylation in the insulin signaling pathway.^33^

Recent studies have shown that intracanal application of MF is effective in treating apical periodontitis in animal models. In a rat model of induced periapical lesions, intracanal MF inhibits disease progression partially through modulation of osteoblast apoptosis.^34^ Furthermore, another experiment has indicated that intracanal MF contributes to the healing of apical periodontitis and is involved in the regulation of the iNOS/NO pathway. MF diminishes LPS induced C–C motif chemokine ligand-2 (CCL-2) secretion from monocytes via inhibition of iNOS/NO production, which plays a major role in apical periodontitis induced by bacteria.^35^

### Drug delivery systems in periodontitis

A major factor governing the efficacy of regenerative scaffolds that encapsulate drugs such as MF is the rate of drug release from these scaffolds.^36^ Most periodontal bone healing occurs during the first 3 months after treatment.^37^ For optimal bone regeneration, slow and sustained release of the growth factors or drugs from the scaffolds is preferable, particularly during the first 3 months.^38^ A triple-layer scaffold design by Chogan et al. successfully releases MF for as many as 12 days into the surrounding tissues.^39^ However, to date, no drug delivery system has been reported to release regenerative drugs or factors in tissues over sufficiently long periods to achieve complete bone regeneration.

### Clinical trials of metformin in periodontitis

Given the important role of MF in bone formation and immunomodulatory function, MF administered locally as an adjunct to scaling and root planning (SRP) has been found to be more useful than SRP alone in treating periodontal defects.^40^ Several clinical trials have reported better treatment outcomes for intrabony defects with the use of various concentrations of MF in the form of a gel.^22^^,^^40^^,^^41^ Pradeep and colleagues, in numerous studies, have demonstrated the adjunct use of 1% MF along with SRP as an effective treatment modality for multiple stages of periodontitis. These studies involved intrabony administration of MF locally after SRP, and have reported better clinical and radiographic parameters than observed in control groups.^42^^,^^45^^,^^47^ In 2017, Kassem et al. have tested synthetically prepared thiolated alginate-based mucoadhesive films of 0.6% MF hydrochloride for intra-pocket local delivery in patients of chronic periodontitis and observed significant improvements in all clinical parameters within the first 6 months.^46^ Use of MF as an adjunct with SRP has also been reported to result in better clinical attachment levels (CAL) and smaller probing depth (PD) than use of topical *Aloe vera* gel (AV) with SRP treatment.^48^ In contrast, 1.2% rosuvastatin (RSV), compared with both 1% MF and placebo, has been found to significantly improve clinical parameters, e.g., decreasing PD and increasing CAL.^49^ In one study, 1% MF has been used in combination with platelet-rich fibrin (PRF) and open flap debridement (OFD) as an intervention with better clinical and radiographic outcomes than MF and OFD alone.^44^ Ample evidence indicates that adjuvant utilization of MF provides an additive benefit to the outcomes of conventional periodontal therapy. The general characteristics of randomized clinical trials of MF on patients with periodontitis are shown in Table 1.

**Table 1:** General characteristics of randomized clinical trials of MF for periodontitis.

	Year	Number of participants	Age (years)	Sex	Study groups and intervention	Main outcomes
Pradeep et al. ^42^	2013	TP = 41 EG I = 10 EG II = 10 EG III = 11 CG = 10	30–50	M = 20 F = 21	EG I = SRP + 0.5% MF EG II = SRP + 1.0% MF EG III = SRP + 1.5% MF CG = SRP + placebo	Better clinical and radiographic parameters were observed in EG than CG. 1% MF showed the highest improvement.
Rao et al.^43^	2013	TP = 50 EG = 25 CG = 25	30–50	M = 50 F = 00	EG = SRP + 1.0% MF CG = SRP + placebo	Better clinical and radiographic parameters were observed in EG than CG.
Pradeep et al.^44^	2015	TP = 120 EG I = 30 EG II = 30 EG III = 30 CG = 30	30–50	M = 60 F = 60	EG I = OFD + PRF EG II = OFD + 1.0% MF EG III = OFD + PRF + 1.0% MF CG = OFD	Better clinical and radiographic parameters were observed in EGs than CG. The PRF+ 1% MF group showed the highest improvement among groups.
Pradeep et al.^45^	2016	TP = 65 EG = 33 CG = 32	25–50	M = 38 F = 27	EG = SRP + 1.0% MF CG = SRP + placebo	Better clinical and radiographic parameters were observed in EG than CG.
Kassem et al.^46^	2017	TP = 20 EG = 10 CG = 10	36–55	M = 10 F = 10	EG = SRP + 0.6% MF multiple layer film CG = SRP + placebo	Better clinical parameters were observed in EG than CG.
Pradeep et al.^47^	2017	TP = 70 EG = 36 CG = 34	30–50	M = 34 F = 30	EG = SRP + 1.0% MF CG = SRP + placebo	Significantly better clinical and radiographic parameters were observed in EG than CG.
Kurian et al.^48^	2018	TP = 90 EG I = 30 EG II = 30 CG = 30	24–42	M = 44 F = 46	EG I = SRP + AV EG II = SRP + 1.0% MF CG = SRP + placebo	Better clinical and radiographic parameters were observed in EGs than CG. Results were significantly improved with the use of 1% MF rather than AV.
Pankaj et al.^49^	2018	TP = 90 EG I = 30 EG II = 30 CG = 30	25–45	M = 44 F = 46	EG I = SRP + 1.2% RSV EG II = SRP + 1.0% MF CG = SRP + placebo	Better clinical and radiographic parameters were observed in EG than CG. Significant improvement was observed in the 1.2% RSV group, as compared with the 1% MF group and CG.

TP, total participants; M, male; F, female; EG, experimental group; CG, control group; MF, metformin; PRF, platelet-rich fibrin; OPD, open flap debridement; AV, *Aloe vera*; RSV, rosuvastatin.

### Role of metformin in oral squamous cell carcinoma

Oral squamous cell carcinoma (OSCC) is a malignant and aggressive tumor of epithelial origin, which is becoming a major cause of cancer-associated death.^50^ Although various treatment modalities, such as radiotherapy, chemotherapy and surgery, are available, none have been demonstrated to be successful in terms of survival rate. For inhibition and identification of the growth of cancer cells, a reliable therapeutic agent is urgently needed.^51^ In the search for a viable agent, MF has shown desirable anti-cancer characteristics.

Owing to its mechanism of action, MF acts as an anticancerous drug both directly and indirectly. It functions directly on cancer-producing cells by activation of AMP-activated protein kinase. By influencing mitochondrial respiration in activation, MF controls energy homeostasis in cells. MF also acts indirectly on host metabolism by decreasing the levels of circulating insulin levels via the phosphoinositide 3-kinase (PI3K) pathway; this action is mediated through an AMPK mediated decrease in gluconeogenesis in the liver.^52^ A decrease in mammalian target of rapamycin complex 1 (mTORC1) activity in pre-malignant cells has been observed, which may diminish the risk of cancer through both AMPK-dependent and independent mechanisms. The mTORC1 complex has been identified to be involved in the pathogenesis of OSCC.^53^ Gutkind et al. have recently performed a clinical trial in patients with oral pre-malignant lesions to assess the effect of MF in OSCC prevention by targeting the phosphoinositide 3-kinase/rapamycin complex 1 (PI3K/mTOR) signaling pathway. MF significantly decreased the conversion of oral pre-malignant lesions in OSCC; moreover, it acted directly on cancer-initiating cells thereby decreasing tumor growth by slowing the activity of mTOR in the mTORC1 complex.^54^ In addition, MF has been reported to increase nerve growth factor receptor–N (NFGR-N) levels by suppressing NFGR-N proteolysis and decreasing p53, thus promoting the anticancer effect.^55^ By inhibiting the expression of yes-assisted protein (YAP), MF suppresses OSCC. A study by Wang et al. has shown the relationship of MF with YAP, an oncoprotein that is encoded by chromosome 11q22 in humans; is responsible for cell proliferation control, regeneration and organ development; and is associated with numerous signaling pathways linked with cancer.^56^

## Regenerative effect of metformin on dental stem cells

The regenerative potential of dental stem cells (DSCs) was first identified in the early 21st century.^57^ Since then, these cells have been found to possess unique characteristics of mesenchymal stem cells (MSCs), such as self-renewal capacity and multilineage differentiation capability with a high rate of proliferation.^58^ DSCs are considered ideal candidates for tissue engineering applications, because they are expandable, have regenerative potential and are more readily obtained than other bone marrow-derived MSCs.

### Effects of metformin on dental pulp stem cells

MF is osteogenic in nature and therefore is suitable to use with DSCs, particularly in combination with dental pulp stem cells, because it enhances bone repair, notably in diabetic conditions in which alveolar bone loss is eminent.^59^ In 2017, Wang and colleagues reported the osteogenic differentiation effect of MF on induced pluripotent stem cell-derived mesenchymal stem cells (iPSC-MSCs) in scaffolds of calcium phosphate cement (CPC). Through activation of the liver kinase B1/AMP-activated protein kinase (LKB1/AMPK) pathway, the drug substantially increases the mineralized nodule formation of iPSC-MSC in scaffolds.^60^ To exploit the osteogenic effects of MF, Gao et al. developed a demineralized dentin matrix as a carrier to target deliver MF and dental pulp stem cells. MF was loaded into the matrix to form a demineralized dentin matrix-MF complex. The seeded dental pulp stem cells in the complex showed better attachment with up-regulation of bone-forming genes, comprising ALP, osteocalcin (OCN), Runx2 and OPN.^61^ In a study by Qin et al., a CPC-chitosan-MF complex was synthesized to complement the osteogenic and odontogenic differentiation of pulp stem cells and showed favorable results.^62^ Another study has used resin containing 20% MF by mass as a model system to induce odontogenic differentiation and later mineral synthesis of dental pulp stem cells. The MF containing resin group showed higher proliferation and mineralization than the control group without MF.^63^ Recently, MF treatment preconditioning has been found to promote the angiogenic ability of pulp stem cell conditioned medium in a dose-dependent manner *in vitro*.^64^

### Effects of metformin on periodontal ligament stem cells

MF has positive effects on periodontal ligament stem cells as well as dental pulp stem cells. In addition to promoting osteogenic differentiation, MF provides protection against oxidative stress-induced damage in periodontal ligament stem cells by activating the protein kinase B/nuclear factor erythroid 2–related factor 2 (Akt/Nrf2) signaling pathway.^65^ A study by Zhang and colleagues has indicated that a 50 μM dose of MF has significant positive effects on proliferation, migration and osteogenic differentiation of PDLSCs *in vitro*.^66^ In a recent study, MF has been used to decipher the involvement of the NPR3-mediated MAPK pathway in increasing the osteogenic differentiation of PDLSCs. Under high glucose conditions via downregulation of NPR3 and inhibition of its downstream MAPK pathway resulted in MF-promoted osteogenic differentiation.^67^ Xu et al. have designed a composite scaffold loading MF composed of β-tricalcium phosphate, chitosan and mesoporous silica by using the freeze-drying method. The synthetically prepared composite scaffold was implanted in alveolar bone defect areas in a rat model of periodontitis. The outcomes were favorable, and these scaffolds supported alveolar bone regeneration.^30^ Together, these results indicate the potential for use of MF in PDLSC-based bone regeneration and periodontal tissue engineering.^68^

### Effects of metformin on stem cells from human exfoliated deciduous teeth

Stem cells from human exfoliated deciduous teeth (SHED) have high potential for use in tissue engineering and regenerative medicine. In 2020, Zhou et al. reported that MF induces osteogenic differentiation of SHEDs by activating the AMPK pathway, and also exerts positive effects on the expression of osteogenic genes and proangiogenic growth factors in SHEDs.^69^ According to a recent study by Deng et al., in addition to promoting cell proliferation and inducing multiple forms of differentiation, MF pre-treatment significantly increases the SHED-mediated angiogenesis *in vivo* of human umbilical endothelial cells; these findings may pave the way to the application of SHEDS pre-treated with MF for tissue regeneration.^70^

## Role of metformin in photodynamic therapy

Photodynamic therapy (PDT) requires the use of photoactive dyes known as photosensitizers, which, after exposure to specific wavelengths, transfer energy to oxygen and form toxic free radical oxygen species. These reactive oxygen species can damage proteins, lipids, nucleic acid, and other molecules.^71^ PDT has potential applications in dentistry in the treatment and diagnosis of infections and cancers of the mouth.^72^ The antimicrobial role of PDT may provide a potential alternative to antibiotics, which are more prone to resistance to oral bacterial flora.^73^ In a study by Pourhajibagher et al., indocyanine green conjugated with nano-curcumin along with MF has been used to form a new photosensitizer with elevated anti-biofilm activity in antimicrobial PDT against *E. faecalis* in root canals under dual wavelength irradiation, thus providing an efficient adjunctive endodontic treatment modality.^74^ Despite evidence from *in vitro* studies in the literature, clinical trials examining the role of MF in endodontics are lacking.

## Role of metformin in osseointegration of implants

Dental implant therapy has emerged as an advantageous treatment modality for the rehabilitation of edentulous patients, owing to its biocompatibility, superior esthetics and association with favorable prognosis.^75^ The success of dental implant therapy is inextricably associated with peri-implant remodeling and osseointegration around implants.^76^ According to previous studies, patients with T2DM have a higher rate of implant failure than healthy controls.^77^ Early osseointegration research has demonstrated that diabetes influences the remodeling of bone around implants. Hyperglycemic conditions can lead to impairment of immune function, inhibition of bone formation, and enhancement of the release of cytokines such as IL-6. Therefore, dental implant osseointegration in patients with diabetes is compromised, thus leading to failure.^78^

In this regard, experiments in animal models have demonstrated that MF monotherapy significantly promotes the osseointegration of endosseous implants.^79^ The effects of MF on wound healing are crucial for implant survival, through countering the adverse effects of advanced glycation end products on osteoblastic cells, including interactions with receptors for advanced glycation end products. As previously discussed, as an AMPK agonist, MF inhibits complex I of the mitochondrial respiratory chain and activates cellular energy metabolism, thereby increasing Runx2 and OPG expression in osteoblasts, decreasing intracellular oxidative stress and the production of advanced glycation end products, and ultimately improving the long term clinical success rate of dental implants.^80^ MF has been demonstrated to increase osseointegration and bone formation in multiple animal models and at numerous doses. Although the extent of the favorable effects has shown mixed results, the findings provide sufficient impetus for studies in human participants in the foreseeable future.^79^, ^80^, ^81^, ^82^, ^83^, ^84^

The general characteristics of animal studies assessing the effect of MF on osseointegration of implants are given in Table 2.

**Table 2:** General characteristics of animal studies assessing the effect of MF on osseointegration of implants.

Author	Year	Animal model	Number of participants	Study groups and intervention	Implant site	Dose of metformin	Study duration	Outcomes
Inouye et al.^79^	2014	Wistar-Kyoto rats Goto-Kakizaki DM rats	EG I = 12 EG II = 12 CG = 12	EG I = DM + MF EG II = DM CG = non-DM (no treatment)	Right maxillary molars	100 mg/kg/day	4 weeks	No significant effect of MF was observed on bone-implant contact and trabecular volume in diabetic rats. MF improved bone volume in diabetic rats.
Serrão et al.^80^	2017	Diabetic rats	EG I = 10 EG II = 10 CG = 10	EG I = DM EG II = DM + MF CG = non-DM (no treatment)	Tibia	100 mg/kg/day	30 days	MF had no significant effect on bone-implant contact and bone area. Increased OPG+ cells and a decreased RANKL/OPG ratio were observed.
Bastos et al.^81^	2017	Wistar rats	EG = 10 CG = 10	EG = MF CG = no treatment	Tibia	40 mg/kg/day	30 days	MF decreased bone-implant contact and bone area. The number of RANKL+ cells increased.
Lin et al. ^82^	2020	Sprague Dawley rats	EG I = 10 EG II = 10 EG III = 10 CG = NS	EG I = sham surgery (n = 10) EG II = OVX + MF EG III = OVX only CG = NS	Right maxillary molars	20 mg/ml/day	14 days	MF enhanced osseointegration and bone formation in rat models of induced osteoporosis.
Yıldırım et al.^83^	2020	Sprague Dawley rats	EG = 10 CG = 10	EG = MF CG = no treatment	Tibia	40 mg/kg/day	28 days	MF induced elevated peri-implant bone fill ratios.
Sun et al.^84^	2021	Wistar rats	EG I = 06 EG II = 06 CG = 06	EG I = MF-50 EG II = MF-100 CG = no treatment	Femur	MF-50: 50 mg/kg/day MF-100: 100 mg/kg/day	4 weeks	MF increased bone-implant contact. At doses of 100 mg/kg/day MF had a greater effect on bone-implant contact than 50 mg/kg/day.

TP, total participants; EG, experimental group; CG, control group; MF, metformin; NS, not specified; OVX, ovariectomy.

## Role of metformin in orthodontics

Another notable aspect of MF use is its ability to induce favorable effects on orthodontic tooth movement. In 2017, Sun and colleagues conducted a study on Wistar rats with induced T2DM to demonstrate the effects of MF on orthodontic movement. Orthodontic appliances were placed in rats in experimental and control groups, and the results were evaluated after 2 weeks. The rats treated with MF exhibited greater tooth movement with normal osteoclast numbers, thus providing histological evidence that MF decreases the risk of undesirable orthodontic tooth movement. Moreover, MF improved immunolocalization of dentin matrix protein and decreased sclerostin expression.^85^

Orthodontic wires undergo corrosion because of the dynamic oral environment and dietary habits after wire clipping. Owing to their non-toxic and environmentally friendly nature, certain pharmaceutical drugs may provide viable alternatives to traditional corrosion inhibitors, including MF.^86^ One such study has assessed the effects of MF in increasing the corrosion resistance of nickel-titanium (Ni–Ti) wires used during orthodontic treatment. Electrochemical studies have been performed to investigate corrosion resistance of orthodontic wire in artificial saliva and have indicated that the corrosion resistance of Ni–Ti wires significantly increases in the presence of MF hydrochloride. The reason for this change remains unclear.^87^

## Limitations

Despite various studies suggesting potential applications of MF in various fields of dentistry, several challenges must be overcome. The long-term efficacy of MF in the treatment of periodontal defects remains to be ascertained. Further research is needed to gauge the long-term efficacy of MF in the management of periodontitis. Limited experimental work has assessed the anti-tumor properties of MF with respect to OSCC, and further studies are required to support its use as a reliable molecular target for therapy. MF has also been shown to increase the osseointegration of dental implants in studies performed in a wide array of animal models, but the dearth of clinical trials in human participants hinders its potential use for ameliorating osseointegration of endosseous implants in patients with diabetes in the near future. Similarly, some evidence in the literature has demonstrated beneficial effects in improving orthodontic tooth movement and resisting orthodontic appliance corrosion in animal models. However, comprehensive studies are required to decipher the mechanism underlying these changes. Regarding the application of MF in stem cell regeneration and photodynamic therapy, the literature has indicated sufficient evidence of favorable effects of MF, but studies have been limited primarily to preclinical cell and animal study models. After demonstration of its numerous positive effects *in vitro* and animal studies, clinical trials in humans should be fast-tracked to assess the extent of the beneficial effects of MF in human participants. Optimum doses should be determined, to maximize potential effects in various applications.

## Conclusion

Given its multiple benefits reported in the literature, MF is a favorable prospect for dental applications. Its osteogenic, regenerative, anti-neoplastic and osseointegration properties have been well explored in recent studies. Although numerous potential applications of MF exist, further long term clinical and animal studies will be critical to assess and explore its efficacy and optimum dosage, and maximize its benefits.

## Source of funding

This research did not receive any specific grant from funding agencies in the public, commercial or not-for-profit sectors.

## Conflict of interest

The authors have no conflicts of interest to declare.

## Ethical approval

No ethical approval is required as it is a review article and no in vivo study has been conducted.

## Authors contributions

MKHU conceptualized and supervised the research and participated in writing the original draft of the article. MSKS, AA, MK, TM, MSM and SN participated in writing and editing the original draft of the article. SU, SB, KSM, ZK and MSZ individually revised the final article. All authors have critically reviewed and approved the final draft, and are responsible for the content and similarity index of the manuscript.
